# CpG Single-Site Methylation Regulates TLR2 Expression in Proinflammatory PBMCs From Apical Periodontitis Individuals

**DOI:** 10.3389/fimmu.2022.861665

**Published:** 2022-03-01

**Authors:** María José Bordagaray, Alejandra Fernández, Jessica Astorga, Mauricio Garrido, Patricia Hernández, Alejandra Chaparro, María Jesús Lira, Peter Gebicke-Haerter, Marcela Hernández

**Affiliations:** ^1^ Laboratory of Periodontal Biology, Faculty of Dentistry, Universidad de Chile, Santiago, Chile; ^2^ Department of Conservative Dentistry, Faculty of Dentistry, Universidad de Chile, Santiago, Chile; ^3^ Faculty of Dentistry, Universidad Andres Bello, Santiago, Chile; ^4^ Department of Periodontology, Faculty of Dentistry, Centro de Investigación e Innovación Biomédica (CIIB), Universidad de Los Andes, Santiago, Chile; ^5^ Department of Orthopedic Surgery, Pontificia Universidad Católica de Chile, Santiago, Chile; ^6^ Institute of Psychopharmacology, Central Institute of Mental Health, Faculty of Medicine, University of Heidelberg, Mannheim, Germany; ^7^ Department of Pathology and Oral Medicine, Faculty of Dentistry, Universidad de Chile, Santiago, Chile

**Keywords:** periapical periodontitis, toll-like receptor 2, CpG island, DNA methylation, RNA messenger, leukocytes, mononuclear

## Abstract

**Introduction:**

Apical periodontitis (AP) is a common oral disease caused by the inflammatory destruction of the periapical tissues due to the infection of the root canal system of the tooth. It also contributes to systemic bacterial translocation, where peripheric mononuclear blood cells (PBMCs) can act as carriers. Toll-like receptor (TLR) 2 mediates the response to infection and activates inflammatory responses. DNA methylation can be induced by bacteria and contributes to the modulation of this response. Despite the evidence that supports the participation of PBMCs in immune-inflammatory disorders, the inflammatory profile and epigenetic regulatory mechanisms of PBMCs in AP individuals are unknown.

**Aim:**

To determine TLR2 gene methylation and inflammatory profiles of PBMCs in AP.

**Methods:**

Cross-sectional exploratory study. Otherwise, healthy individuals with AP (n=27) and controls (n=30) were included. PMBCs were isolated by a Ficoll gradient, cultured for 24 hours, and both RNA and DNA were extracted. DNA was bisulfite-treated, and specific sites at the promoter region of the TLR2 gene were amplified by qPCR using validated primers. To verify its amplification, agarose gels were performed. Then, the PCR product was sequenced. mRNA expression of TLR2 was determined by qPCR. The soluble levels of 105 inflammatory mediators were first explored with Proteome Profiler Human Cytokine Array Kit. Consequently, tumor necrosis factor (TNF)-α, interleukin (IL)-6, IL-10, IL-6Rα, IL-1β, and IL-12p70 levels were measured by Multiplex assay.

**Results:**

PBMCs from individuals with AP demonstrated a proinflammatory profile showing higher soluble levels of TNF-α, IL-6, and IL-1β compared to controls (p<0.05). Higher TLR2 expression and higher global methylation pattern of the promoter region of the gene were found in AP compared to controls (p<0.05). The CpGs single-sites at positions -166 and -146 were completely methylated, while the site -102 was totally unmethylated, independently of the presence of AP. DNA methylation of CpG single-sites in positions -77 and +24 was positively associated with TLR2 expression.

**Conclusions:**

PBMCs from AP subjects show a hyperinflammatory phenotype and TLR2 upregulation in association with single CpG-sites’ methylation from the TLR2 gene promoter, thereby contributing to a sustained systemic inflammatory load in individuals with periapical endodontic diseases.

## Introduction

Apical periodontitis (AP) is among the main causes of tooth loss and is typified by the inflammatory destruction of the periapical tissues as a consequence of the infection of the root canal system of the tooth (1). Its hallmark is the development of an apical lesion of endodontic origin (ALEO) (2), and it has been identified as an independent contributor to low-grade systemic inflammation, and a non-classical risk factor for non-communicable diseases (2).

The pathogenesis of AP is mediated by the host-pathogen interactions, in which, toll-like receptors (TLR) play a pivotal role. TLRs are a family of receptors activated by damage-associated molecular patterns (DAMPs) and pathogen-associated molecular patterns (PAMPs) (3). Among them, TLR2 classically recognizes lipoteichoic acid from Gram-positive bacteria (4), but it also is involved in the recognition of lipopolysaccharide from Gram-negative bacteria (5, 6). For this reason, oral pathogens like *Porphyromonas (P.) gingivalis* and *P. endodontalis*, can trigger the TLR2 pathway (7, 8). The interaction TLR2-ligand activates intercellular signaling that induces nuclear factor *κ*-B (NF*κ*B) translocation to the nucleus and the consequent inflammatory cascade activation (9). The expression of TLR2 can be regulated by transcriptional changes that depend on epigenetic modifications in ALEOs (10).

Epigenetics can regulate the activation of the immune cell in response to infection by transcriptional reprogramming (11, 12). These modifications are characterized as being stably inherited to the cell progeny without altering the DNA sequence. Epigenetic modifications involve histone post-translational changes, microRNA interference, and DNA methylation (13). DNA methylation, the addition of a methyl group on the fifth carbon of a DNA cytosine residue (14), is often found in rich C and G dinucleotide (CpG) regions and can be induced by bacteria to either allow pathogen persistence or promote host defense (11). Gene silencing is the most frequent regulatory mechanism associated with DNA hypermethylation, but a dual regulatory function has also been proposed (15). TLR2 contains a CpG island being highly susceptible to epigenetic regulation. It is also overexpressed at local oral tissues in periodontitis (16) and AP (10). The higher expression of TLR2 in ALEOs is associated with a hypomethylated DNA pattern of the promoter region and specific CpG single-sites, supporting the epigenetic regulation in AP (10).

Recent evidence sustains that AP courses with low-grade systemic inflammation (2, 17); moreover, endodontic bacteria and/or their products, such as *P. endodontalis*, can invade the peri radicular tissues and translocate to distant sites within peripheric blood mononuclear cells (PBMCs) (18). In this context, the internalization of bacteria or their antigens might result in mononuclear cell activation *via* TLR2 recognition (19), and reasonably contribute to a higher systemic inflammatory burden. Despite the evidence that supports the participation of PBMCs in systemic immune-inflammatory diseases, such as rheumatoid arthritis or cardiovascular diseases (20–23), the behavior and regulatory mechanisms of PBMCs in AP are currently unknown. In this study, we aimed to determine the TLR2 gene methylation and inflammatory profiles of PBMCs in AP.

## Materials and Methods

### Study Design

This analytic cross-sectional exploratory study was conducted under the approval of the Ethics-Scientific Committee of the Central Metropolitan Health Service (Number 2017/70) and from the Faculty of Dentistry, Universidad de Chile (Number 2016/08). All the volunteers were informed about the objectives and procedures of the study and signed the informed consent. All procedures followed the ethical standards of the institutional and/or national research committees and were under the 1964 Helsinki declaration and its later amendments.

### Study Participants

Otherwise healthy individuals aged between 18-40 years old consulting at the Dental Clinic, Faculty of Dentistry, Universidad de Chile were enrolled between 2016-2020 if they had a clinical diagnosis of primary AP (1) and no concomitant systemic diseases. The volunteers had one or more teeth with a clinical diagnosis of asymptomatic (AAP) or symptomatic apical periodontitis (SAP). The diagnosis was confirmed by a negative response to pulp sensitivity tests, the absence or presence of pain to dental percussion, respectively (1), and the presence of ≥ 1 ALEO equal to or greater than 3 mm in diameter equivalent to a periapical index ≥ 3 (24). Controls met the same criteria in the absence of AP. Exclusion criteria were severe periodontitis, pregnancy, body mass index ≥ 30 kg/m^2^, and antibiotic and/or anti-inflammatory drug consumption three months before the study (2).

### Clinical Evaluation of Participants

General health assessment and medical history were conducted by the endodontist and a trained nurse technician. Age, sex, educational level, smoking habit, and classic cardiovascular risk factors (25, 26), were recorded in pre-established charts. Individuals with a history of non-communicable diseases were excluded from the study and referred to a medical center for further evaluation.

Oral clinical evaluations were carried out by a qualified general dentist (C.V.), a periodontist (P.H.), an endodontist (M.G). The ALEO size was determined as the mean of vertical and horizontal diameters (mm). Full-mouth radiographic evaluations were obtained. Intraoral parameters including periodontal probing depth (PPD) and clinical attachment levels (CAL) were determined with a periodontal probe (UNC-15, Hu-Friedy, USA) in six sites per tooth. All the enrolled patients received hygiene instruction, full-mouth ultrasonic scaling, and coronal prophylaxis before entering the study. The subjects with AP diagnosis were properly treated (M.G) after the sample collection.

### Samples

Fasting blood samples were obtained from AP (n=27) and healthy controls volunteers (n=30). The samples were collected by venipuncture of the antecubital vein by a qualified technician (27). Fractions of the collected blood samples were submitted to the clinical laboratory of the University of Chile Hospital for the determination of glycated hemoglobin levels (HbA1c) and lipid profiles. The rest of the samples were submitted and processed in the Laboratory of Periodontal Biology for isolation of PBMCs using Ficoll-Paque Premium 1.073 (GE Healthcare^®^) according to the manufacturer’s instructions.

### Profiling of Secreted Inflammatory Proteins

Isolated PBMCs from individuals with AP as well as controls were cultured in a 96-well plate in a 2 x 10^5^ cell density and incubated at 37°C and 5% CO_2_ in RPMI-1640 medium. After 24 hours, the supernatants were recovered and the inflammatory profile of PBMCs first was explored by a commercial Proteome Profiler Human Cytokine Array Kit, identifying 105 soluble inflammatory proteins simultaneously (R&D system Inc., Minneapolis, USA). Briefly, samples were mixed with a cocktail of biotinylated detection antibodies fixed on a membrane-based sandwich immunoassay, according to the manufacturer’s instructions. Three independent samples per group were loaded in duplicates. The captured proteins were visualized by chemiluminescence and semi quantification was performed by densitometric scanning (Bruker MI SE, Billerica, MA, USA). The intensity was determined by the mean pixel density of the duplicate spots that represent each cytokine. The negative control was the clear area of the membrane. Also, the levels of tumor necrosis factor (TNF)-*α*, interleukin (IL)-1*β*, IL-6, IL-6R*α*, IL-12p70, and IL-10 were measured by Multiplex assay (R&D System Inc., Minneapolis, USA) using a Luminex platform (Milliplex MAGPIX^®^ System, Merck Millipore, Massachusetts, USA), following the manufacturers’ recommendations. The data were analyzed by the MILLIPLEX AnalystR software (v5.1, Viagene Tech, Carlisle, MA, USA).

### DNA and RNA Isolation

DNA was purified from PBMCs using the DNeasy isolation kit (QIAGEN Inc., Valencia, CA, USA) and RNA was extracted with TRIzol Reagent (Invitrogen™), following the respective manufacturer’s instructions. The quality and concentration of DNA and RNA were quantified using a spectrophotometer (Bio-Tek, Winooski, VT, USA) in the ratio of 260:280. A reverse transcription kit (Thermo Fisher Scientific, Carlsbad, CA, USA) was used for synthesizing the single-stranded cDNA from the RNA samples (8 *μ*l).

### mRNA Expression Level

The mRNA expression levels of TLR2 were determined by the amplification of 50 ng of cDNA by qPCR through KAPA SYBR^®^ FAST qPCR Kits (KAPA Biosystems), using the forward sequence 5’-CTCTCGGTGTCGGAATGTC-3’ and the reverse sequence 3’-AGGATCAGCAGGAACAGAGC-5’, as previously reported (10). Amplification was performed at 95°C for 30 s, 40 cycles at 95°C for 30 s, and 60°C for 30 s. Additionally, a melting curve was run from 60 to 95°C to identify the presence of non-specific products. Gene expression was normalized by 18S ribonucleic RNA expression levels in controls and calculated by the 2–ΔΔCt method (28).

### DNA Methylation Analysis

Purified DNA (200 ng) from each sample was bisulfite-treated (EZ DNA Methylation-Direct™ Kit, Zymo Research Irvine, CA, USA). Bisulfited DNA was amplified by KAPA SYBR^®^ Fast qPCR Kits (KAPA Biosystems, Woburn, MA, USA) with validated primer set for TLR2 promoters CpG island: forward sequence 5’-TAAGGGCGGGAGTTTGTTGGGAAGTAC-3’ and reverse sequence 3’-CTCCGAACCGACCTACCCGAAACTAAA-5’, as previously reported (10). The selected primers for original or bisulfited DNA were obtained from Gene Bank: http://www.ncbi.nlm.nih.gov/nuccore/NC_000004.12?report=genbank&from=153684256&to=15370609 by Methprimer program (http://www.urogene.org/cgi-bin/methprimer/methprimer.cgi). The PCR amplified product integrity was determined on agarose gels (2%) and submitted to Sanger sequencing service (Macrogen, Seoul, Korea). The sequencing results were analyzed with the BiQ Analyzer program and expressed as the relative frequency of global methylation (methylated CpG sites/the total sites) and single-CpG methylated sites within the CpG island from the TLR2 gene promoter.

### Statistical Analysis

Data distribution of numerical variables was assessed by the Shapiro-Wilk test. Comparisons between AP and controls were analyzed by *t*-test or Mann-Whitney U-test according to data distribution. Spearman’s correlation was used to evaluate the association between quantitative variables. The comparison of categorical variables was performed with the chi-square test. To explore the associations between TLR2 global and CpG single-site’s methylation, as independent variables, and TLR2 mRNA expression as the outcome variable independently of potential confounders, we ran linear bivariate and multiple regression models using backward stepwise regression, removing the covariates with p ≥ 0.2. To explore the influence of periapical inflammation and other potential exposure factors on the global methylation of the TLR2 gene promoter´s CpG island, we performed bivariate fractional regression for potential confounders and then fit the models including those covariates with significant p values (p<0.1). To facilitate interpretation, we estimated and presented the marginal effects. Statistical analyses were performed with Stata statistical package 12 (Stata-Corp. LP, TX, USA). All figures were constructed on GraphPad Prism 9 software (Graph-Pad Software Inc., San Diego, CA, USA).

## Results

**Figure 1:**
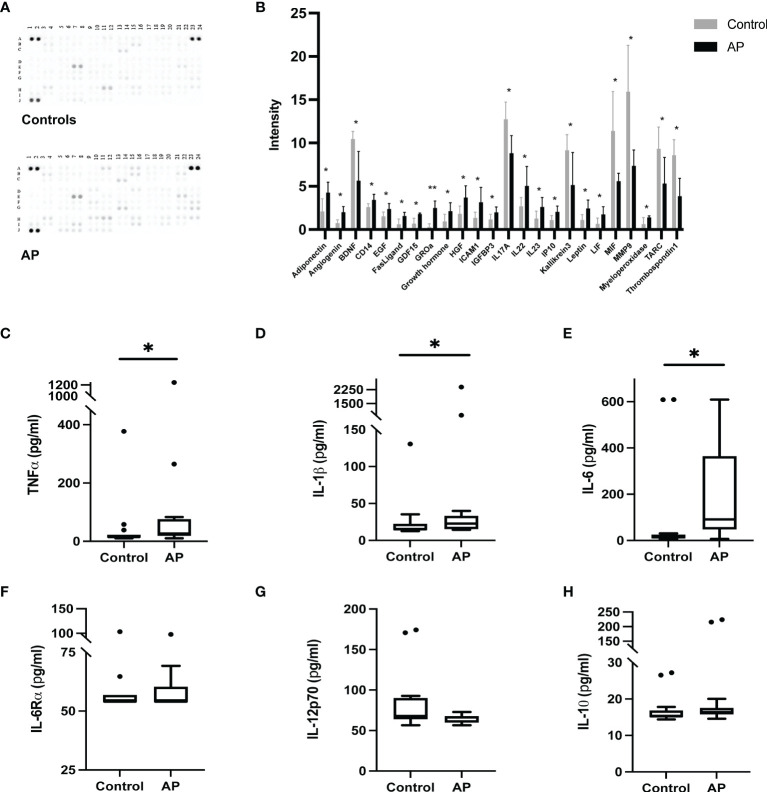
Profiling of secreted inflammatory proteins in PBMCs from apical periodontitis and control individuals. **(A)** Inflammatory protein profile **(B)** Relative levels of inflammatory proteins. Levels of **(C)** TNF-*α*, **(D)** IL-1*β*, **(E)** IL-6 **(F)** IL-6R*α*, **(G)** IL-12p70 and **(H)** IL-10 expressed in pg/ml. AP apical periodontitis. *p < 0.05, **p < 0.001.

General, intraoral, and serum parameters of enrolled volunteers are shown in **Table 1**. Age, sex, and smoking habit were higher with AP than controls (p<0.05), while the rest of the variables were evenly distributed among groups. The secreted inflammatory profile of PBMCs is shown in **Figure 1**. A pro-inflammatory phenotype (**Figures 1A, B** **)** composed of 17 up-regulated and 7 down-regulated inflammatory mediators was determined in AP versus controls (p<0.05). Also, the concentrations (pg/mL) of inflammatory cytokines commonly associated with systemic inflammation were determined (**Figures 1C–H**). In AP the levels of the pro-inflammatory cytokines TNF-*α*, IL-1*β*, and IL-6 were significantly higher than controls (p=0.029, p=0.032, p=0.017; respectively). On the other hand, the levels of IL-6R*α*, IL-12p70, and IL-10 did not show differences between the study groups (p=0.555, p=0.426, p=0.117; respectively).

**Table 1:** Sociodemographic, general, oral, and serum parameters of study individuals with apical periodontitis (AP) and healthy controls.

	Controls (n=30)	AP (n=27)	p value
Age (years)^#^	22 (6)	25 (15)	**0.02**
Females (n,%)	15 (50%)	21 (77.8%)	**0.03**
Smokers (n,%)	5 (17.2%)	13 (48.1%)	**0.01**
Educational level	3	3	0.59
High blood pressure (n, %)	2 (7.4%)	1 (3.3%)	0.49
PD (mm)^##^	2.05 (0.43)	2.08 (0.56)	0.34
CAL (mm)^##^	1 (0.83)	1 (1.36)	0.12
Number of apical lesions^##^	—	1.14 (0.4)	—
Apical lesions size (mm)^#^	—	8 (6.5)	—
Total cholesterol (mg/dL)^#^	161 (53)	160 (62)	0.54
LDL cholesterol (mg/dL)^#^	83 (37)	84.4 (49.8)	0.70
HDL cholesterol (mg/dL)^#^	56 (16)	54 (21)	0.23
Triglycerides (mg/dL)^#^	92 (60)	121 (97)	0.88
HbA1c^##^	5.1 (0.16)	5.2 (0.2)	0.09

AP, apical periodontitis; PD, probing depth; CAL, clinical attachment level; LDL, low-density lipoprotein; HDL, high-density lipoprotein; HbA1c, glycated hemoglobin; ^#^median (interquartile range); ^##^mean(standard deviation); **bold**: p < 0.05.

**Figure 2:**
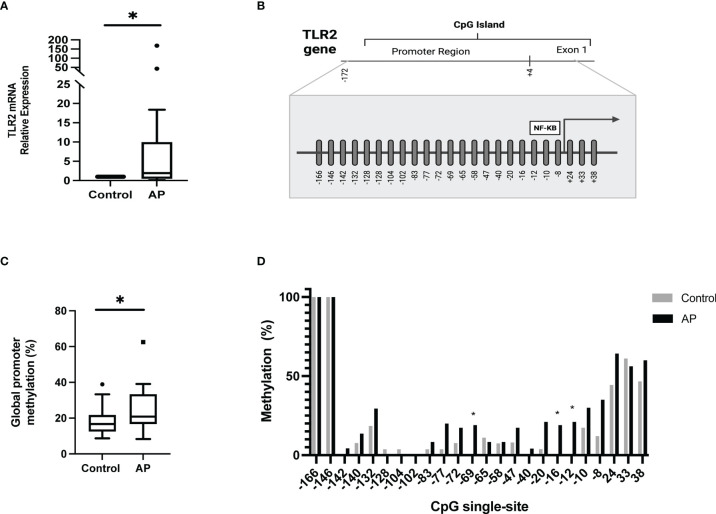
Expression levels and methylation profile of TLR2 in PBMC from apical periodontitis and control individuals. **(A)** mRNA relative expression of TLR2. The expression data were normalized to 18S rRNA and relative to controls, using the differences between 2–ΔΔCT. *p = 0.013. **(B)** Schematic representation of the human CpG single-sites from TLR2 gene promoter. **(C)** Global promoter methylation frequency (%). *p=0.043 **(D)** CpG single-site methylation frequency (%). AP apical periodontitis.

Analysis of TLR2 expression revealed significantly higher mRNA expression levels in AP subjects compared to the control group (**Figure 2A**; p=0.013). The association between TLR2 gene expression and TNF-α, IL-1β, IL-6, IL-6Rα, IL-12p70 and IL-10 levels resulted in moderate and low correlations (r= 0.34, 0.16, 0.37, 0.19, -0.22, 0.24, respectively; p>0.05). The epigenetic regulation of the TLR2 gene was explored by determining the methylation status of the CpG island at the gene promoter, localized between positions -166 to +38, as shown in **Figure 2B**. A higher global methylation pattern of the gene promoter was found in AP, compared to healthy controls (p=0.043) with medians (interquartile range) of 20.8% (15.3) and 16.7% (9.2), respectively (**Figure 2C**). The methylation frequencies of the CpG single-sites are shown in **Figure 2D**. While the sites -166 and -146 were completely methylated, the site -102 was totally unmethylated, independently of the presence of the periapical disease; the rest of the CpG single-sites on the other hand were highly variable regarding their methylation status.

To further understand the influence of the TLR2 promoter’s methylation status over its transcriptional activity, linear regression modeling was performed (**Table 2** **)**. The global methylation status alone did not associate with TLR2 transcriptional activity (p=0.42). At the site level, CpG methylated positions at -77 and +24 were positively associated with TLR2 transcriptional activity in bivariate models (p=0.036 and 0.034, respectively) and multivariate model (p=0.005 and 0.014, respectively), and the later model explained 33% of the variability.

Table 2Influence of the TLR2 promoter’s methylation status in gene expression.

**Table:** 

A. Global methylation status		
Variable	Ln TLR2 mRNA relative expression	
	(N = 50)	
	Coef ± SE	p-value
Global methylation (%)	-0.02 ± 0.019	0.42
R-squared	0.01	

Coef, coefficient; SE, standard error; **bold:** p < 0.05.

**Table:** 

B. CpG single-sites		
Site	Ln TLR2 mRNA relative expression	
	Coef ± SE	p-value
CpG -77^1^	1.35 ± 0.63	**0.036**
R^1^	0.095	
CpG +24^1^	1.05 ± 0.47	**0.034**
R^1^	0.141	
CpG -77^2^	2.15 ± 0.71	**0.005**
CpG +24^2^	1.11 ± 0.42	**0.014**
R-squared^2^	0.332	

^1^Bivariate (CpG -77 N=46; CpG +24 N=32); ^2^Multivariate Model (N=31), including both covariates shown. Coef, coefficient; SE, standard error; **bold:** p < 0.05.

Finally, potential modifying factors of TLR2 promoter’s gene methylation were explored (**Table 3**). Among all variables, the periapical diagnosis and aging were identified to influence the global methylation of the TLR2 gene promoter. Bivariate analysis showed that the global methylation was increased by the presence of ALEO (p=0.022) and aging (p=0.0004). However, when including both covariates in the multivariate model, only aging remained associated. The global methylation was increased by +12 percentage points by each year (p=0.011). Also, age correlated positively with the promoter´s global methylation (rho= 0.44; p=0.0012).

**Table 3:** Modifiers of TLR2 promoter’s methylation status.

Exposure variables	Fractional point change (CI 95%)	p-value
PA Diagnosis (ALEO)^1^	0.03 (0.002 - 0.06)	**0.022**
Age (years)^1^	0.15 (0.06 - 0.23)	**0.0004**
PA Diagnosis (ALEO)^2^	0.02 (-0.01 – 0.05)	0.162
Age (years)^2^	0.12 (0.02 – 0.21)	**0.011**

^1^Bivariate model. ^2^Multivariate model using periapical (PA) diagnosis and age as covariates. N = 51. CI, Confidence Interval; **bold:** p < 0.05.

## Discussion

AP has largely been considered as a local infection that causes the inflammation and disruption of the periapical tissues, though recent evidence demonstrates that ALEOs also contribute to the systemic bacterial and inflammatory burden, acting as a non-classic risk factor for non-communicable diseases (2, 18). In the present study, we show for the first time that PBMCs present a pro-inflammatory phenotype and overexpress TLR2 in AP individuals in association with the gene promoter’s methylation.

PBMCs, which include monocytes, natural killers, and T and B lymphocytes, are key cells in the preservation of systemic homeostasis and defense against infection. Furthermore, their cytokine production has been proposed to reflect the activity of several immune-inflammatory diseases (20–23). Our results showed that PBMCs from AP individuals have a global proinflammatory secretory profile with significantly higher levels of TNF-*α*, IL-1*β*, and IL-6 in comparison with healthy controls. Despite there being no reports in AP, PBMCs from individuals with periodontitis have also shown a proinflammatory profile with enhanced secretion of interferon-**γ**, IL-6, IL-12p70, IL-17, and monocyte chemoattractant protein-1 versus their healthy counterparts (29). In line with this, PBMCs from patients with periodontitis stimulated *in vitro* with LPS of *Escherichia coli*, have also demonstrated a proinflammatory secretory pattern with higher levels of the cytokine’s TNF-α and IL-6, among others, in comparison with PBMCs from healthy individuals (30). TNF-α and IL-6 are associated with pro-inflammatory and bone resorptive local responses (31, 32), as well as C-reactive protein (CRP) synthesis, which is involved in systemic inflammation. Moreover, higher plasma levels of these cytokines have been associated with the development of cardiovascular events (33). In our study, the higher levels of TNF**-**α, IL-1β, and IL-6 in PBMCs from AP individuals were also accompanied by the upregulation of several cardiovascular disease biomarkers like adiponectin and angiogenin (34). Overall, global PBMCs analysis revealed an active proinflammatory state in PBMCs from AP individuals, thus contributing to the inflammatory burden and its long-term consequences. Further evaluation of the specific cell subsets contributing to this inflammatory phenotype will contribute to unveil systemic inflammation associated with oral infections.

TLR2 plays an important role in the recognition of microbial stimuli and the triggering of innate and adaptive immune responses. The local expression of TLR2 in periapical tissues has been associated with the presence and progression of AP. It has been demonstrated that TLR2 expression increases along with the development and progression of ALEOs at different time points (35). TLR2 is also overexpressed in human ALEOs particularly in symptomatic AP, which can represent the active form of the disease, compared to asymptomatic AP and healthy tissues (10). Regarding TLR2 in the systemic context, the evidence supports that TLR2 is upregulated in PBMCs of individuals with inflammatory non-communicable diseases (36, 37). Also, the transcription of this pattern recognition receptor in PBMCs is downregulated after periodontal therapy in periodontal disease individuals (38). These data agree with our results where we showed that TLR2 is overexpressed in PBMCs from AP individuals in comparison with controls, supporting the role of ALEOs in PBMC activation.

DNA methylation, one of the main epigenetic modulating mechanisms, has been reported to regulate several chronic inflammatory pathologies (12, 39, 40). The CpG islands are highly susceptible to methylation reprogramming. In the case of the TLR2 gene, it comprises the promoter region, exon 1, and part of intron 1 (41). Most available evidence supports that TLR2 expression and the methylation status of the gene promoter have an inverse relation (10, 42–44). However, our study does not support similar findings in PBMCs from AP individuals. In line with our results, the expression levels of TLR2 were independent of the gene’s methylation pattern in the HUVEC cell line stimulated with TNF-α (45). Also, the methylation status of the TLR2 promoter’s region showed no differences among normal and inflamed human pulp tissue (46), despite the upregulation of TLR2 having been previously reported (47). Overall, TLR2 DNA methylation might cause activation or repression depending on the stimulus (48, 49) in a tissue/cell-specific manner (50, 51).

Emerging evidence supports that DNA methylation can also act by regulating the binding of transcription factors (TF) or methyl-CpG-binding proteins in a CpG site-specific manner (52–54). Particularly in ALEOs, unmethylated CpG single-sites -10 and -12 are associated with transcriptional upregulation of TLR2 (10). Interestingly, these CpG single-sites are close to the NF*κ*B binding sequence. Accordingly, when unmethylated they allow the TF binding and gene transcription (41). In our study, the methylation of CpG single-sites -77 and +24 in PBMCs was positively associated with TLR2 mRNA expression. Despite there being no previous reports from TLR2, methylated CpG single-sites have also been associated with the upregulation of gene expression. FGFR3 gene expression demonstrated a positive correlation between CpG single-sites promoters’ methylation in uterine corpus endometrial carcinoma and breast invasive carcinoma (55). The mechanisms supporting a direct association between DNA methylation and transcription upregulation remain to be elucidated, but it has been proposed that methylated CpG single-sites could create new binding sites for TFs favoring the initiation of transcription activity (56).

A few studies sustain that aging is associated with global hypermethylation of CpG islands (57). In our study the methylation of TLR2 promoter in PBMCs was influenced by aging, being the main factor of the higher global methylation in AP in comparison with controls. The link between aging and hypermethylation is complex and multivariable, but it has been proposed that it could be attributed to the accumulation of stochastic methylation events (58) and the down-regulation of ten-eleven translocation demethylases (59).

Concluding, PBMCs show a hyperinflammatory phenotype and TLR2 upregulation in AP, which in turn, is influenced by the methylation of single CpG-sites from the TLR2 gene promoter, contributing to a sustained systemic inflammatory load in individuals with periapical endodontic diseases.

## Data Availability Statement

The original contributions presented in the study are included in the article/supplementary material. Further inquiries can be directed to the corresponding author.

## Ethics Statement

The studies involving human participants were reviewed and approved by the Ethics-Scientific Committee of the Central Metropolitan Health Service (N 2017/70) and from the Faculty of Dentistry, Universidad de Chile (N2016/08). The patients/participants provided their written informed consent to participate in this study.

## Author Contributions

MH, MG, AC, and PG-H: conceptualization and design. MH, MB, and ML: data curation. MH: Funding acquisition. MH, AF, and MB: Formal analysis. MB, AF, and JA: laboratory experiments. MH: Project administration. MB and MH: design the figures. MH: Resources. MH: Software. MG, MH, and PH: Supervision. MB, AF, and MH: Writing manuscript. All authors critically reviewed and approved the manuscript.

## Funding

This study was funded by FONDECYT grants 1160741 and 1200098 from ANID, the Chilean government. MB is a recipient of the scholarship ANID 21210551, from the Chilean Government. AF is a recipient of the scholarship CONICYT 21181377, from the Chilean Government. MG is a recipient of the scholarship ANID 21210509, from the Chilean Government.

## Conflict of Interest

The authors declare that the research was conducted in the absence of any commercial or financial relationships that could be construed as a potential conflict of interest.

## Publisher’s Note

All claims expressed in this article are solely those of the authors and do not necessarily represent those of their affiliated organizations, or those of the publisher, the editors and the reviewers. Any product that may be evaluated in this article, or claim that may be made by its manufacturer, is not guaranteed or endorsed by the publisher.

## References

[1] Gutmann JL Baumgartner JC Gluskin AH Hartwell GR Walton RE. Identify and Define All Diagnostic Terms for Periapical/Periradicular Health and Disease States. J Endod (2009) 35(12):1658–74. doi: 10.1016/j.joen.2009.09.028 19932340

[2] Garrido M Cárdenas AM Astorga J Quinlan F Valdés M Chaparro A. Elevated Systemic Inflammatory Burden and Cardiovascular Risk in Young Adults With Endodontic Apical Lesions. J Endodontics (2019) 45(2):111–5. doi: 10.1016/j.joen.2018.11.014 30711165

[3] Kono H Rock KL. How Dying Cells Alert the Immune System to Danger. Nat Rev Immunol (2008) 8(4):279–89. doi: 10.1038/nri2215 PMC2763408 18340345

[4] Takeuchi O Hoshino K Kawai T Sanjo H Takada H Ogawa T. Differential Roles of Tlr2 and Tlr4 in Recognition of Gram-Negative and Gram-Positive Bacterial Cell Wall Components. Immunity (1999) 11(4):443–51. doi: 10.1016/s1074-7613(00)80119-3 10549626

[5] Hirschfeld M Weis JJ Toshchakov V Salkowski CA Cody MJ Ward DC. Signaling by Toll-Like Receptor 2 and 4 Agonists Results in Differential Gene Expression in Murine Macrophages. Infect Immun (2001) 69(3):1477–82. doi: 10.1128/IAI.69.3.1477-1482.2001 PMC98044 11179315

[6] Kirikae T Nitta T Kirikae F Suda Y Kusumoto S Qureshi N. Lipopolysaccharides (Lps) of Oral Black-Pigmented Bacteria Induce Tumor Necrosis Factor Production by Lps-Refractory C3h/Hej Macrophages in a Way Different From That of Salmonella Lps. Infect Immun (1999) 67(4):1736–42. doi: 10.1128/iai.67.4.1736-1742.1999 PMC96522 10085012

[7] Tang Y Sun F Li X Zhou Y Yin S Zhou X. Porphyromonas Endodontalis Lipopolysaccharides Induce Rankl by Mouse Osteoblast in a Way Different From That of Escherichia Coli Lipopolysaccharide. J Endod (2011) 37(12):1653–8. doi: 10.1016/j.joen.2011.08.015 22099899

[8] Harokopakis E Albzreh MH Martin MH Hajishengallis G. Tlr2 Transmodulates Monocyte Adhesion and Transmigration *Via* Rac1- and Pi3k-Mediated Inside-Out Signaling in Response to Porphyromonas Gingivalis Fimbriae. J Immunol (2006) 176(12):7645–56. doi: 10.4049/jimmunol.176.12.7645 16751412

[9] de Oliviera Nascimento L Massari P Wetzler L. The Role of Tlr2 in Infection and Immunity. Front Immunol (2012) 3:79. doi: 10.3389/fimmu.2012.00079 22566960 PMC3342043

[10] Fernández A Veloso P Astorga J Rodríguez C Torres VA Valdés M. Epigenetic Regulation of Tlr2-Mediated Periapical Inflammation. Int Endod J (2020) 53(9):1229–37. doi: 10.1111/iej.13329 32426871

[11] Bierne H Hamon M Cossart P. Epigenetics and Bacterial Infections. Cold Spring Harb Perspect Med (2012) 2(12):a010272. doi: 10.1101/cshperspect.a010272 23209181 PMC3543073

[12] Qin W Scicluna BP van der Poll T. The Role of Host Cell DNA Methylation in the Immune Response to Bacterial Infection. Front Immunol (2021) 12:696280. doi: 10.3389/fimmu.2021.696280 34394088 PMC8358789

[13] Zhang L Lu Q Chang C. Epigenetics in Health and Disease. Adv Exp Med Biol (2020) 1253:3–55. doi: 10.1007/978-981-15-3449-2_1 32445090

[14] Laird PW. Principles and Challenges of Genomewide DNA Methylation Analysis. Nat Rev Genet (2010) 11(3):191–203. doi: 10.1038/nrg2732 20125086

[15] Lyko F. The DNA Methyltransferase Family: A Versatile Toolkit for Epigenetic Regulation. Nat Rev Genet (2018) 19(2):81–92. doi: 10.1038/nrg.2017.80 29033456

[16] Rojo-Botello NR García-Hernández AL Moreno-Fierros L. Expression of Toll-Like Receptors 2, 4 and 9 Is Increased in Gingival Tissue From Patients With Type 2 Diabetes and Chronic Periodontitis. J Periodontal Res (2012) 47(1):62–73. doi: 10.1111/j.1600-0765.2011.01405.x 21848608

[17] Georgiou AC Crielaard W Ouwerling P McLean W Lappin DF van der Waal SV. The Influence of Apical Periodontitis on the Concentration of Inflammatory Mediators in Peripheral Blood Plasma and the Metagenomic Profiling of Endodontic Infections: Study Design and Protocol. Contemp Clin Trials Commun (2021) 21:100686. doi: 10.1016/j.conctc.2020.100686 33490705 PMC7810621

[18] Bordagaray MJ Fernández A Garrido M Astorga J Hoare A Hernández M. Systemic and Extraradicular Bacterial Translocation in Apical Periodontitis. Front Cell Infect Microbiol (2021) 11:649925. doi: 10.3389/fcimb.2021.649925 33816354 PMC8017189

[19] Sahingur SE Xia XJ Alamgir S Honma K Sharma A Schenkein HA. DNA From Porphyromonas Gingivalis and Tannerella Forsythia Induce Cytokine Production in Human Monocytic Cell Lines. Mol Oral Microbiol (2010) 25(2):123–35. doi: 10.1111/j.2041-1014.2009.00551.x PMC4017325 20331800

[20] Barth S Kleinhappl B Gutschi A Jelovcan S Marth E. *In Vitro* Cytokine Mrna Expression in Normal Human Peripheral Blood Mononuclear Cells. Inflamm Res (2000) 49(6):266–74. doi: 10.1007/pl00000206 10939616

[21] Bomprezzi R Ringnér M Kim S Bittner ML Khan J Chen Y. Gene Expression Profile in Multiple Sclerosis Patients and Healthy Controls: Identifying Pathways Relevant to Disease. Hum Mol Genet (2003) 12(17):2191–9. doi: 10.1093/hmg/ddg221 12915464

[22] Corbi SCT de Vasconcellos JF Bastos AS Bussaneli DG da Silva BR Santos RA. Circulating Lymphocytes and Monocytes Transcriptomic Analysis of Patients With Type 2 Diabetes Mellitus, Dyslipidemia and Periodontitis. Sci Rep (2020) 10(1):8145. doi: 10.1038/s41598-020-65042-9 32424199 PMC7235087

[23] Sørensen LK Havemose-Poulsen A Bendtzen K Holmstrup P. Aggressive Periodontitis and Chronic Arthritis: Blood Mononuclear Cell Gene Expression and Plasma Protein Levels of Cytokines and Cytokine Inhibitors. J Periodontol (2009) 80(2):282–9. doi: 10.1902/jop.2009.080347 19186969

[24] Ørstavik D Kerekes K Eriksen HM. The Periapical Index: A Scoring System for Radiographic Assessment of Apical Periodontitis. Dental Traumatol (1986) 2(1):20–34. doi: 10.1111/j.1600-9657.1986.tb00119.x 3457698

[25] Chalmers J MacMahon S Mancia G Whitworth J Beilin L Hansson L. 1999 World Health Organization-International Society of Hypertension Guidelines for the Management of Hypertension. Guidelines Sub-Committee of the World Health Organization. Clin Exp Hypertens (1999) 21(5-6):1009–60. doi: 10.3109/10641969909061028 10423121

[26] Grundy SM Becker D Clark T Cooper RS Denke MA Howard J. Executive Summary of the Third Report of the National Cholesterol Education Program (Ncep) Expert Panel on Detection, Evaluation, and Treatment of High Blood Cholesterol in Adults (Adult Treatment Panel III). Jama (2001) 285(19):2486–97. doi: 10.1001/jama.285.19.2486 11368702

[27] Fouad AF Barry J Caimano M Clawson M Zhu Q Carver R. Pcr-Based Identification of Bacteria Associated With Endodontic Infections. J Clin Microbiol (2002) 40(9):3223–31. doi: 10.1128/JCM.40.9.3223-3231.2002 PMC130810 12202557

[28] Livak KJ Schmittgen TD. Analysis of Relative Gene Expression Data Using Real-Time Quantitative Pcr and the 2(-Delta Delta C(T)) Method. Methods (2001) 25(4):402–8. doi: 10.1006/meth.2001.1262 11846609

[29] Basic A Serino G Leonhardt Å Dahlén G Bylund J. The Secretion of Cytokines by Peripheral Blood Mononuclear Cells of Patients With Periodontitis and Healthy Controls When Exposed to H2s. J Oral Microbiol (2021) 13(1):1957368. doi: 10.1080/20002297.2021.1957368 34408814 PMC8366616

[30] De Oliveira NF Andia DC Planello AC Pasetto S Marques MR Nociti FH Jr.. Tlr2 and Tlr4 Gene Promoter Methylation Status During Chronic Periodontitis. J Clin Periodontol (2011) 38(11):975–83. doi: 10.1111/j.1600-051X.2011.01765.x 21899586

[31] Wajant H Pfeffer K Pfizenmaier K Scheurich P. Tumor Necrosis Factors in 1998. Cytokine Growth Factor Rev (1998) 9(3-4):297–302. doi: 10.1016/s1359-6101(98)00013-6 9918127

[32] Boyce BF Li P Yao Z Zhang Q Badell IR Schwarz EM. Tnf-Alpha and Pathologic Bone Resorption. Keio J Med (2005) 54(3):127–31. doi: 10.2302/kjm.54.127 16237274

[33] Ryan S Taylor CT McNicholas WT. Systemic Inflammation: A Key Factor in the Pathogenesis of Cardiovascular Complications in Obstructive Sleep Apnoea Syndrome? Thorax (2009) 64(7):631–6. doi: 10.1136/thx.2008.105577 19561283

[34] Dhingra R Vasan RS. Biomarkers in Cardiovascular Disease: Statistical Assessment and Section on Key Novel Heart Failure Biomarkers. Trends Cardiovasc Med (2017) 27(2):123–33. doi: 10.1016/j.tcm.2016.07.005 PMC5253084 27576060

[35] Barreiros D Pucinelli CM Oliveira KMH Paula-Silva FWG Nelson Filho P Silva L. Immunohistochemical and Mrna Expression of Rank, Rankl, Opg, Tlr2 and Myd88 During Apical Periodontitis Progression in Mice. J Appl Oral Sci (2018) 26:e20170512. doi: 10.1590/1678-7757-2017-0512 29995146 PMC6025885

[36] Chang H Zhang QY Lin Y Cheng N Zhang SQ. Correlation of Tlr2 and Tlr4 Expressions in Peripheral Blood Mononuclear Cells to Th1- and Th2-Type Immune Responses in Children With Henoch-Schönlein Purpura. Int J Clin Exp Med (2015) 8(8):13532–9. PMC4612976 26550291

[37] Iwahashi M Yamamura M Aita T Okamoto A Ueno A Ogawa N. Expression of Toll-Like Receptor 2 on Cd16+ Blood Monocytes and Synovial Tissue Macrophages in Rheumatoid Arthritis. Arthritis Rheum (2004) 50(5):1457–67. doi: 10.1002/art.20219 15146415

[38] Papapanou PN Sedaghatfar MH Demmer RT Wolf DL Yang J Roth GA. Periodontal Therapy Alters Gene Expression of Peripheral Blood Monocytes. J Clin Periodontol (2007) 34(9):736–47. doi: 10.1111/j.1600-051X.2007.01113.x PMC2670555 17716309

[39] Fernández A Cárdenas AM Astorga J Veloso P Alvarado A Merino P. Expression of Toll-Like Receptors 2 and 4 and Its Association With Matrix Metalloproteinases in Symptomatic and Asymptomatic Apical Periodontitis. Clin Oral Investig (2019) 23(12):4205–12. doi: 10.1007/s00784-019-02861-9 30806798

[40] Huang KT Chen YC Tseng CC Chang HC Su MC Wang TY. Aberrant DNA Methylation of the Toll-Like Receptors 2 and 6 Genes in Patients With Obstructive Sleep Apnea. PloS One (2020) 15(2):e0228958. doi: 10.1371/journal.pone.0228958 32069296 PMC7028278

[41] Haehnel V Schwarzfischer L Fenton MJ Rehli M. Transcriptional Regulation of the Human Toll-Like Receptor 2 Gene in Monocytes and Macrophages. J Immunol (2002) 168(11):5629–37. doi: 10.4049/jimmunol.168.11.5629 12023360

[42] Walsh SW Chumble AA Washington SL Archer KJ Sahingur SE Strauss JF. 3rd. Increased Expression of Toll-Like Receptors 2 and 9 Is Associated With Reduced DNA Methylation in Spontaneous Preterm Labor. J Reprod Immunol (2017) 121:35–41. doi: 10.1016/j.jri.2017.05.003 28622534 PMC5533291

[43] Furuta T Shuto T Shimasaki S Ohira Y Suico MA Gruenert DC. DNA Demethylation-Dependent Enhancement of Toll-Like Receptor-2 Gene Expression in Cystic Fibrosis Epithelial Cells Involves Sp1-Activated Transcription. BMC Mol Biol (2008) 9:39. doi: 10.1186/1471-2199-9-39 18423053 PMC2387165

[44] Benakanakere M Abdolhosseini M Hosur K Finoti LS Kinane DF. Tlr2 Promoter Hypermethylation Creates Innate Immune Dysbiosis. J Dental Res (2014) 94(1):183–91. doi: 10.1177/0022034514557545 PMC4270813 25389002

[45] Diesel B Ripoche N Risch RT Tierling S Walter J Kiemer AK. Inflammation-Induced Up-Regulation of Tlr2 Expression in Human Endothelial Cells Is Independent of Differential Methylation in the Tlr2 Promoter Cpg Island. Innate Immun (2012) 18(1):112–23. doi: 10.1177/1753425910394888 21768203

[46] Cardoso FP de Faria Amormino SA Dutra WO Ribeiro Sobrinho AP Moreira PR. Methylation Pattern of the Cd14 and Tlr2 Genes In Human Dental Pulp. J Endodontics (2014) 40(3):384–6. doi: 10.1016/j.joen.2013.11.024 24565657

[47] Mutoh N Tani-Ishii N Tsukinoki K Chieda K Watanabe K. Expression of Toll-Like Receptor 2 and 4 in Dental Pulp. J Endod (2007) 33(10):1183–6. doi: 10.1016/j.joen.2007.05.018 17889686

[48] Zardo G Fazi F Travaglini L Nervi C. Dynamic and Reversibility of Heterochromatic Gene Silencing in Human Disease. Cell Res (2005) 15(9):679–90. doi: 10.1038/sj.cr.7290337 16212874

[49] Ching TT Maunakea AK Jun P Hong C Zardo G Pinkel D. Epigenome Analyses Using Bac Microarrays Identify Evolutionary Conservation of Tissue-Specific Methylation of Shank3. Nat Genet (2005) 37(6):645–51. doi: 10.1038/ng1563 15895082

[50] Yan Y Dalmasso G Nguyen HT Obertone TS Charrier-Hisamuddin L Sitaraman SV. Nuclear Factor-Kappab Is a Critical Mediator of Ste20-Like Proline-/Alanine-Rich Kinase Regulation in Intestinal Inflammation. Am J Pathol (2008) 173(4):1013–28. doi: 10.2353/ajpath.2008.080339 PMC2543070 18787102

[51] Kao YH Chen YC Cheng CC Lee TI Chen YJ Chen SA. Tumor Necrosis Factor-Alpha Decreases Sarcoplasmic Reticulum Ca2+-Atpase Expressions *Via* the Promoter Methylation in Cardiomyocytes. Crit Care Med (2010) 38(1):217–22. doi: 10.1097/CCM.0b013e3181b4a854 19730253

[52] Barros SP Offenbacher S. Modifiable Risk Factors in Periodontal Disease: Epigenetic Regulation of Gene Expression in the Inflammatory Response. Periodontol 2000 (2014) 64(1):95–110. doi: 10.1111/prd.12000 24320958

[53] Fürst RW Kliem H Meyer HH Ulbrich SE. A Differentially Methylated Single Cpg-Site Is Correlated With Estrogen Receptor Alpha Transcription. J Steroid Biochem Mol Biol (2012) 130(1-2):96–104. doi: 10.1016/j.jsbmb.2012.01.009 22342840

[54] Rishi V Bhattacharya P Chatterjee R Rozenberg J Zhao J Glass K. Cpg Methylation of Half-Cre Sequences Creates C/Ebpα Binding Sites That Activate Some Tissue-Specific Genes. Proc Natl Acad Sci (2010) 107(47):20311. doi: 10.1073/pnas.1008688107 21059933 PMC2996703

[55] Spainhour JCG Lim HS Yi SV Qiu P. Correlation Patterns Between DNA Methylation and Gene Expression in the Cancer Genome Atlas. Cancer Inf (2019) 18:1176935119828776. doi: 10.1177/1176935119828776 PMC6376553 30792573

[56] Zhu H Wang G Qian J. Transcription Factors as Readers and Effectors of DNA Methylation. Nat Rev Genet (2016) 17(9):551–65. doi: 10.1038/nrg.2016.83 PMC5559737 27479905

[57] Fraga MF Agrelo R Esteller M. Cross-Talk Between Aging and Cancer: The Epigenetic Language. Ann N Y Acad Sci (2007) 1100:60–74. doi: 10.1196/annals.1395.005 17460165

[58] Christensen BC Houseman EA Marsit CJ Zheng S Wrensch MR Wiemels JL. Aging and Environmental Exposures Alter Tissue-Specific DNA Methylation Dependent Upon Cpg Island Context. PloS Genet (2009) 5(8):e1000602. doi: 10.1371/journal.pgen.1000602 19680444 PMC2718614

[59] Jessop P Toledo-Rodriguez M. Hippocampal Tet1 and Tet2 Expression and DNA Hydroxymethylation Are Affected by Physical Exercise in Aged Mice. Front Cell Dev Biol (2018) 6:45. doi: 10.3389/fcell.2018.00045 29732371 PMC5922180

